# Breast Cancer and Screening Prevention Programmes: Perceptions of Women in a Multicultural Community in Southern Thailand

**DOI:** 10.3390/ijerph20064990

**Published:** 2023-03-12

**Authors:** Dusanee Suwankhong, Pranee Liamputtong, Tum Boonrod, Witchada Simla, Sermsak Khunpol, Sasithorn Thanapop

**Affiliations:** 1 Department of Public Health, Thaksin University, Pa Phayom 93210, Phatthalung, Thailand; 2 College of Health Sciences, VinUniversity, Hanoi 100000, Vietnam; 3 Department of Library, Information Science and Communication Arts, Thaksin University, Muang 90000, Songkhla, Thailand; 4 Master of Public Health Programme, School of Public Health, Walailak University, Thasala 80160, Nakhon-Si-Thammarat, Thailand; 5 Research Center of Data Science for Health Science, Walailak University, Thasala 80160, Nakhon-Si-Thammarat, Thailand

**Keywords:** breast cancer, perceptions, screening prevention, risk group, rural community, qualitative study

## Abstract

Background: Breast cancer is a leading cause of morbidity and mortality among women worldwide and in Thailand. Objective: To explore perceptions of breast cancer and screening prevention programmes among a group of at-risk women in a multicultural setting in southern Thailand. Methods: Semi-structured in-depth interviews were used for data collection with 30 at-risk group women. Women from Muslim and Buddhist backgrounds were purposively included in this study. The thematic analysis method was used to analyse the data. Results: Four themes were identified from our data: perceptions of breast cancer, being diagnosed with breast cancer and anxiety, stigma: effects of breast cancer, and breast self-screening and prevention of breast cancer. The participants had some knowledge about the risk factors for breast cancer. However, participants perceived that breast cancer could occur to individual women at any time and that it was not possible to entirely prevent the disease, even when following a breast self-examination programme. However, most participants perceived that whether one would be afflicted by breast cancer depended also on Allah and their own karma. All participants were encouraged to attend breast self-screening training by healthcare providers of local health centres, but they had no confidence to perform self-screening soon after finishing the training programme. This became the reason for a lack of regular self-screening with responsibility left to health practitioners. Although participants were aware that breast self-screening should be their routine practice, there were multiple barriers to this, including accurate knowledge about breast cancer, belief, self-awareness, screening skills and healthcare facilities. Breast self-screening was recognised as an important means of early detection. However, most women did not perform this regularly, which could increase their risk of developing breast cancer. Conclusions: Public health providers need to be more concerned about the perceptions, beliefs and practices regarding breast cancer and develop prevention practices that work better for women living in more diverse cultural locations so that they may be able to follow preventive practices and reduce their vulnerability to breast cancer.

## 1. Introduction

Cancer today causes higher morbidity and mortality rates worldwide compared with previous decades and this is likely to continue in the future [1,2,3]. Cancer has become the first or second top cause of death before 70 years of age in many countries [4]. Among cancer cases, breast cancer is recognised as an important public health problem: it has become the most common disease among women worldwide, with a high number annually of new cases and deaths globally [1,5,6]. The World Health Organization has confirmed that breast cancer has been the most common cancer in recent years, accounting for nearly 2.3 million cases in 2020 [7].

In Thailand, according to the National Cancer Institute, breast cancer incidence was reported to be about 40 per cent, and was the top disease diagnosed among Thai women. Although the rate of breast cancer was relatively high among 45–50-year-old women, there was also a high incidence of breast cancer among younger women. In southern Thailand (where our study was conducted), the Thai National Cancer Institute reported that these were about 26 per cent of the total breast cancer cases in 2019 [8].

Breast cancer is caused by various factors, including postmenopausal age, late menopause, physical inactivity, undertaking hormone treatment, including estrogen and progestin, and even the use of alcohol [2,5]. Family history has been found to be an important risk factor, particularly in first-generation family members compared to those who had no history of family members being diagnosed with breast cancer [5]

To prevent the advance of illness and to reduce either morbidity or mortality rates, breast cancer screening plays a crucial role in ensuring early detection [9,10,11]. Early detection and appropriate healthcare can be important strategies for reducing the burden of breast cancer. There are several techniques for breast cancer screening which suit individuals and social contexts. These include self-examination of breasts and mammogram practices [12]. In our previous study [12], breast self-examination was perceived by women as an important skill that could help detect abnormal signs from the earliest stages. However, the literature suggests that many at-risk women do not pay sufficient attention to such screening programmes due to many barriers in their lives [12,13,14]. Some at-risk groups have less opportunity to access screening services provided by the healthcare sector. This could increase the risk of developing breast cancer among them compared with those who can access such services [12].

Most women may have a good understanding that breast screening can prevent the severity of breast cancer. However, many still lack knowledge about correct screening skills and do not receive regular screening and care by healthcare providers, whilst some may not make it a priority in their daily lives. This is partly because of the influence of socio-cultural factors [15]. In some societies, women may not know much about breast cancer; this can lead to overlooking the risk of developing it, its symptoms, and not knowing how to prevent it [12,16]. In some societies, fear and shame relating to breast health examination, as well as fear of healthcare facilities and the outcomes of screening, remain major issues that prevent women from accessing screening programmes [9]. When visiting health centres, women may experience many difficulties that cause them to ignore the significance of screening for breast cancer. For example, the location of a health facility may be too far from their home [17,18].

In Thailand, the Ministry of Public Health has promoted breast cancer screening vigorously since 2015 and continues to emphasise it as the main policy for reducing mortality rates. Comprehensive strategies have been combined in practice focusing on proactive breast cancer screening programmes by public health workers and educating at-risk groups to follow self-breast examination practices that will lead to early detection. However, the incidence rate of breast cancer in Thailand is still showing an increasing trend, as noted above [8].

The study on which this paper is based examined the perception of breast cancer and prevention practices among women living in a multicultural community in southern Thailand. It was undertaken because little is known about this issue in this part of Thailand. This paper discusses perceptions of breast cancer and screening prevention experiences among a group of at-risk women in a particular setting. Thus, the findings from our study could be beneficial in terms of tailoring education in a culturally sensitive way to promote more practical interventions for at-risk groups of women living in rural environments, thereby promoting the uptake of breast cancer screening and helping women to avoid breast cancer.

### Health Beliefs Model (HBM): A Theoretical Framework

The health beliefs model was adopted for this study because the model can help to predict people’s behaviour based on whether they desire to pursue health promotion activities [19]. This model has three main components: modifying factors, individual beliefs, and action. Modifying factors focus mainly on the socioeconomic background of individuals (i.e., age, gender, education, and ethnicity). Individual beliefs deals with perceived susceptibility, perceived severity of diseases, perceived benefits, perceived barriers, and perceived self-efficacy. The action component deals with individual behaviours regarding outcomes. To achieve certain behaviours, cues to action play an important role as they trigger action. This can be internal (such as the feeling that a symptom increases perceived threats) or external (such as publicity by media, medical advice, and information received while visiting a health centre or listening to a friend’s diagnosis). In particular, perceived susceptibility to and the severity of the disease is related to how individuals perceive a threat. However, each component in the health belief model is linked to the others and predicts health behaviour practice [19,20,21].

Our paper uses these six dimensions to examine the perception of breast cancer and prevention practice on the part of a group of at-risk women in southern Thailand. It seeks to understand how these women perceived their susceptibility to breast cancer, the severity of the disease, the benefits they gained, the barriers they faced, and their self-efficacy beliefs regarding maintaining healthy breasts [19]. An individual’s perceived benefits, barriers and self-efficacy are predictable factors that encourage them to maintain or prevent following such practices [22]. If low barriers are perceived, this is associated with positive behaviour toward breast cancer prevention [23]. Thus, the findings of this study provide current information about prevention practice among a group of women at risk in rural areas. This might help to reduce women’s chances of developing breast cancer and lead to longer and healthy lives among women in rural regions of the country.

## 2. Materials and Methods

This study adopted a descriptive qualitative design to gain a deep understanding of perceptions and screening prevention practices for breast cancer among risk group women in a multicultural community in southern Thailand. Semi-structured in-depth interviews were used to collect data [24]. In-depth interviews allowed the researchers to converse with the participants in their natural settings in a relaxed atmosphere to obtain in-depth information from the participants.

### 2.1. Ethical Approval

This study obtained ethical approval from the Thaksin University Ethics Committee Thailand (COA No. TSU 2021-013 REC. No. 0252).

### 2.2. Participants and Recruitment

Thirty women who met the criteria of the study were purposively invited to share their perceptions and beliefs about breast cancer and screening. Purposive sampling involves selecting potential participants who meet the criteria of the study [24]. The inclusion criteria included: (1) women living in a multicultural area, (2) aged from 30 to 59 years old, (3) free from breast cancer diagnosed during the data collection period, and (4) being happy to participate in this study. The exclusion criteria were leaving the community for the duration of data collection. To recruit the participants, we contacted healthcare providers working in a local healthcare setting in the community to direct us to participants who met the study criteria. A snowball sampling method was also employed to expand the number of potential participants in the study. The method is used extensively in research involving hard-to-reach people [24].

More than one-third of the participants in this study were aged between 40 and 49 (40.00%) or had obtained a primary school degree (33.33%). More than two-thirds were Muslim (70.00%). The majority were married (83.33%). Agriculture was the main occupation among these women (66.67%). Two-thirds had no family history of any kind of cancer (60.00%). The majority had no history of breast problems (86.67%) The most usual method used for screening was breast self-examination. More than two-thirds of participants still experienced normal menstruation (73.33%). For those whose menstruation had stopped, the age when it stopped was most often over 50 (61.54%). The characteristics of the study participants are shown in Table 1.

### 2.3. Data Collection

We started carrying out data collection after obtaining ethics clearance from the university ethics committee. Before the interview, we made an appointment via village health volunteers who were responsible for participants’ healthcare in the study areas a day before confirming the appointed time and place for conversation. Most participants preferred to have the face-to-face conversation at the community centres located in the central part of the village where they felt more relaxed. Written informed consent was obtained before the interview started. The first author, who is a native speaker living in the southern region, asked several open-ended questions to each participant. These included: (1) how do you perceive breast cancer? (2) what would be the chance for women to develop breast cancer in a lifetime? (3) what would you do if you were diagnosed with breast cancer? and (4) can breast cancer be prevented? How? Notes were taken during the conversation to remind the interviewer to probe further.

All interviews were conducted by the first author. She used the local language (southern Thai dialect) for conversation to allow women to be more comfortable and able to articulate their perceptions and experiences related to the study objectives with more ease. Each interview lasted between one and one and a half hours and was recorded using a digital voice recorder. Interviews were continued until little new data emerged, indicating that saturation had occurred [24]. A small amount of compensation was provided to individual participants for their time.

### 2.4. Data Analysis

All interviews were transcribed in the Thai language to maintain the original meaning. A thematic analysis method was used for data analysis [24]. Open coding was initially undertaken by reading the transcript line-by-line several times. This allowed us to develop familiarity with the data from the interview. We did not use any computer software, but relied on a manual analysis strategy using highlighter pens to emphasize key codes. Later, the codes were grouped into categories. Axial coding was then applied to combine similar categories to form a higher level of categories and themes. Finally, selective coding was adopted to group conclusive themes enabling development of the core story of the findings. We ended the process of thematic data analysis when there were very few new themes that could be constructed.

In addition, the rigor of the study was ensured by using the member checking method. As soon as each interview was transcribed, the first author checked the transcripts with study participants for accuracy prior to analysis of the data. After the coding and themes were completed, these were used as a basis for discussion with co-researchers. All translated verbatim quotes used in the paper were cross-checked with the second author who is a bi-cultural researcher and proficient in both Thai and English. These strategies helped to ensure the credibility of the data.

## 3. Results

Four themes were constructed from our data: perceptions of breast cancer, being diagnosed with breast cancer and anxiety, stigma: effects of breast cancer, and breast self-screening and prevention of breast cancer.

### 3.1. Perceptions of Breast Cancer

All participants in this study perceived breast cancer as a very serious illness. If a person got the disease, she would never be cured. She would live with many sufferings in life and die soon after being diagnosed, although there was advanced medical technology and the availability of modern medicine for treatment. Most women stated that cancer intruded on a patients’ organs and lymph nodes, leading to abscesses and infection of affected organs. They remarked that the lymph node was a susceptible part for the development of breast cancer and the lymph node was the food supply of cancer. It also quickly expanded to other organs to increase the severity of the disease. Breast cancer was perceived by participants as *pai heab* or *pai ngeab* (a silent killer) as it did not show any obvious sign in advance, unlike other diseases. However, most would know when it had destroyed cells and mammary glands, which would be too late for them to prevent breast cancer. Kan, a Buddhist woman, mentioned:

*It is a very scary disease for all women who have this disease. It does not have any warning signs. We know when it comes to the third or fourth stage. There are no warning signs like coughing, so we ignore any abnormal signs. It means we just have to have our breasts cut out. Even though the breast was removed, it cannot be healed. We will die only.*

Both Buddhist and Muslim women stated that breast cancer was caused by various factors: genetics, environment, food consumption and individual behaviour. Many Buddhist participants stated that genetics was the main cause of breast cancer. If women had a breast cancer history in the family, they would have a higher chance of developing this disease compared to others who had no family history. The environmental conditions where they lived were recognised as contributing factors to breast cancer. From the perspective of many Muslim participants, nowadays people faced many kinds of pollution and chemical substances around them because they were living in the modern world. For example, there were many cars on the road, factory industry, chemicals used in agricultural areas, and in construction sites, which increased the risk of vulnerability to disease and illness. The food consumption behaviour of the individuals was the main contributing factor recognised by these participants. They pointed out that modern society encouraged people and young generations to consume grilled food, processed food, oily meal, and inorganic vegetables and meat. People no longer followed traditional ways of living, which relied very much on natural resources for living to promote good health. Kanee, a Muslim woman, said:

*If having genetic breast cancer, our generation and the next would have a high chance of getting this disease. The environment around us affects our lives. We get pollution from cars on the road. There are lots of fast food and chemical ingredients, you see. In the past, we didn’t have things like this. There were not many cars and we didn’t inhale pollution and gas like today.*

Some Muslim women articulated that breast cancer would occur among women who breastfeed. They explained that the breast had very soft tissue consisting of blood and lymph, so it was very susceptible to bruises, abscesses and infections. During breastfeeding, the breast would be bumped, and it would get bruised easily from touching and sucking. Later the tissue and cell around the breast could become decomposed and turn into cancer. They suggested that having discharge and purulence coming out from the nipple was an obvious symptom of breast cancer and these signs signified the development of breast cancer in the long run. More than one-third of Buddhist women participants reported that women who breastfed could get pain around the breast. The feeling of pain meant that the pathogen was intruding into blood and lymph systems. It would damage cells and tissue and could lead to breast cancer one day. Kanya, a Buddhist woman said:

*We have never known until we see blood and purulence come out from the nipples…then we can realise that we are the victim of breast cancer It is a very scary and serious condition for women, you see. No one wants this.*

### 3.2. Being Diagnosed with Breast Cancer and Anxiety

All women hoped that they would not experience breast cancer, although they understood that, by the nature of living, that one would have a mix of *tuk* (difficulty) and *suk* (happiness) in life. Life was unpredictable. Individuals might not be affected by cancer, but they might have an accident or chronic illness. So, it was important for them to have awareness of their own breast health. Both Buddhist and Muslim women believed that disease, illness, or unhealthy conditions could occur to anyone at any time. It was difficult to know what disease would come to one and when it would disrupt the body. A Buddhist woman, Kayanee, said: “*Life is uncertain, we never know and we have to tum jai (accept it). We could die by an ageing process, not because of any diseases. It is much better like this and it is okay to think like this*”.

Many Muslim women believed that whether one would have breast cancer or not depended on Allah or Muhammad. They believed that illness was one of the means that Allah used to test human patience for his people. However, if one always acted with good deeds, and even if a woman was diagnosed with breast cancer, she would get a less serious condition and have a better chance of recovery. This is the nature of the human life cycle. Ka pee pointed out: “…*Allah has given us life and he would control our health, life, and future…if he gave you breast cancer, you will get it one day. Believe me, we cannot avoid it*”. Kama remarked:

*Sometimes Allah just wants to test us to whether we have tolerance or are strong enough to live. Getting disease is somewhat the way to encourage people to develop the skill of dealing with suffering when facing life-threatening things. If some persons can accept [breast cancer diagnosis], if one can live with it, she is seen as strong woman or a patient who can lead a daily life. We have to accept what happened to our lives.*

For Thai Buddhist women, breast cancer was caused by one’s karma, referred to as *kam* in Thai. Due to her bad actions in the past, and if she had this disease in her previous life, a woman would not be able to avoid getting this disease again in this life and even the next life. Aree expressed her belief:

*…as a Buddhist person, I believed that if we have to face breast cancer in life, it is because I was afflicted by this disease in the past life…it might follow me to the next life thought because of good deeds (kam dee) and bad deeds (kam choa).*

Women perceived that breast cancer created suffering and uncertainty in life. Their lifestyles, in particular, would completely change. Many agreed that, if they were afflicted by breast cancer, their lives would be controlled by medicine and healthcare providers until the last minute of life. However, some stated that they had to continue their lives without being worried too much because the illness was a part of being a human being. They should understand and lead a normal life like others who did not have breast cancer. Mama expressed: “*…if one is diagnosed with breast cancer, her life becomes miserable. But we have to live with hope -try to live like others…*”.

### 3.3. Stigma: Effects of Breast Cancer

Stigma was an important effect of breast cancer raised by most women in the study. As the breast is perceived to be a symbolic part of being a woman, if one was diagnosed with breast cancer, it might be necessary that their breasts be removed, and this would affect their femininity. Many women imagined their future appearance and beauty as a result of a mastectomy operation. They pointed out that a person with breast cancer would undergo treatment and mastectomy and it was a common treatment they had seen. One would need to seek ways to restore breast shape and keep a good appearance as a woman which could create stress among them. Samami, a Buddhist participant, shared her thoughts: “*If being diagnosed with breast cancer, it is difficult to dress beautifully like others. The breasts will not be in a balanced shape. We have to think about how to fix the problem and it is a stressful thing. There are more burdens in life and we would have worries later on*”.

Losing breasts was a great concern, especially among those who had babies, as they would be unable to breastfeed their babies. They would feel guilty about this since they could not perform a good mothering role like others. Kaja, a Muslim woman stated: “*Losing breast, even one side, how can we breastfeed children? It will be a shame if cannot give him breastmilk*. ” A Buddhist participant also added: “*Our breast is the best source of breastmilk and baby needs breastmilk. It is the nature of women to give breastmilk and we cannot do this if we have no breasts…it causes us not to be able to do a good mothering role*”.

However, many pointed out that having breast cancer nowadays was not a stigmatised condition as women would receive empathy and support from others. The nature of being a woman would not have disappeared although they had one or no breasts. So, they could continue their normal life as it was. Sane, a Muslim woman, said: “*It is not stigmatised at all…just our health is not the same. But one can live a normal life, although living with just one breast*”.

All participants pointed out that the nature of living in a rural setting where people had close relationships with others, including their relatives and neighbors, allowed them to visit and support each other as part of their daily lives. When community members faced difficulties, they received support to enable easier living and encouragement to continue their lives. The participants shared that this unique character of mutual support would develop more during difficult times for community members. Kaja remarked: “*Our community forms good culture. We help each other and care for each other. We give mental support and wish them for recovery when [one] faces a hard time in life.*” Rana, a Buddhist woman, added: “*We will visit if one gets illness for sure. We don’t leave them behind. Even during the COVID-19 outbreak, we would visit and at least give some support in different forms (money, food, stuff) and this helps*”.

### 3.4. Breast Self-Screening and Prevention of Breast Cancer

Breast screening was seen by most women who participated in our study as an important means to detect breast cancer and reduce the severity and complications of the disease. They understood that screening could detect abnormal signs earlier which would allow them to seek proper treatment and have a higher chance of recovery compared to those who had never followed screening practices. Samon, a Buddhist, pointed out: “*Yes, if detecting quickly, it can be treated quickly. Some persons may not check their breasts regularly, they might get disease easily*”. However, a few women felt anxious if they had to perform self-breast screening as they might detect some abnormal signs, and this would lead to fears. Thus, they avoided touching their breasts. Samami said: “*I am afraid of breast cancer and try not to think about it including worry if [I] have to do breast self-examination. If I did it, [I] fear of feeling the lump*”.

All women prevented ill health by engaging in low-risk behaviours for developing breast cancer, such as consuming organic products and growing their own vegetables. They tried to adopt positive behaviors, such as avoiding crowded communities as it created stress and pollution, keeping away from smoking, and taking raw food. They believed that following such positive practices could reduce the possibility of breast cancer development.

However, individuals’ beliefs and perceptions could be barriers to regular screening. Several participants thought that breast cancer could not be prevented. They believed that life was unpredictable, and people would never know what would happen to them in their everyday lives. Ka Sha, a Muslim participant, said: “*like a bottle, I can use a lid to cover and it can prevent bugs or ants from getting into the bottle, but breast cancer is not like that. We don’t know when germs will intrude into our cells or which route, so how we can prevent it? Thus, although we knew that breast screening is important, it does not always help*”. She also elaborated:

*How can we prevent it because it tends to show signs when it has seriously infected and no way to cure it, just has to do mastectomy only … If part of our body is damaged or infected, it has to follow proper treatment.*

Many younger women did not believe that they would have breast cancer in life. They perceived that breast cancer was found only in older women. From their experience, this disease tended to occur among women aged between 50 and above. Thus, they assumed that they would have a low risk of getting breast cancer and did not pay much attention to the prevention of breast cancer. They did not search for any information about breast cancer screening programmes and practices. Yanee, a Muslim woman, stated: “*I have never done self-breast screening properly. The disease has not happened yet and it is very rare to occur in younger women like us*.” Apinya, a Buddhist woman, stated:

*[I] am just 33 years old now and have not thought about it. Most [breast cancer] is found in older women, so I never search for information about breast cancer and have never been interested to look for such information. [I] don’t know how to do breast screening. [I] have never thought I will get this disease as I am still young.*

All participants understood that screening was a basic technique for the early detection of breast cancer. Training sessions and follow-up activities were provided by healthcare professionals once a year. Many women pointed out that they could follow screening procedures during the training activity, but they did not feel confident when they had to do it on their own at home. They wanted healthcare providers to check their breasts more than once a year because this was the best means to determine whether they had the risk of developing breast cancer or not. They remarked that, if they were left to do it on their own, it might be too late to detect the disease at an early stage because they were not confident if they performed the right detection technique or not. Kaja said: “*[I] am not confident at all. I just touch slightly like this while showering and cannot feel anything I am a bit worried though.”* Nee further stated:

*Healthcare providers in the local area teach us how to do self-screening. They said we can do it while showering, but [I] don’t know how deep or hard we have to press on the breast. Sometimes, we did not press hard enough and stop when feeling pain. so, I am not sure if I perform it correctly or not.*

Some participants paid little attention to breast health promotion and prevention. This is because they did not believe that they would get breast cancer. They then overlooked information and knowledge about breast health screening, campaigns, and prevention activities. As they had to attend to their everyday life activities and responsibilities, they saw disease prevention and self-care promotion as a future priority.

Importantly, many women, particularly Muslim women, felt shame to be screened by healthcare staff because they had to unclothe while screening. They considered the breast as a private part of individuals. Rana, a Muslim woman, stated: “*I feel embarrassed. I have to open my breast naked. I am terrified of showing my private organ. It is bud see (embarrassment or sham)*”. But Siri, a Buddhist woman, pointed out: “*I am not so shy. I am not a young woman and have three children. It also has a curtain while screening. Then it’s okay*.”

Many participants informed us that they had no concerns about breast screening because no family members had been diagnosed with any kind of cancer before. However, for those who had family members with cancer, this led them to have more concern about screening for early detection. They spent time checking their health and performed breast cancer screening more often. Wanna who just recently had a father diagnosed with cancer pointed out: “*At the beginning, I have not thought about breast detecting. I do concern more when my father was diagnosed with cancer. I check it more often and touch if any lymph around breast*”.

## 4. Discussion

The majority of women who participated in this study perceived that there were many risk factors causing breast cancer: genetics, individual health behaviour, family member history, age of menopause, history of abnormal breasts and environment, for example. Individuals could experience breast cancer at any time during their lifetime as a result of these factors. This is in accordance with Morman et al. [2] who suggested that the risk of breast cancer is caused by varied factors and affected by individual contexts. This includes such factors as age, history of the family, age at menopause, age when first giving birth and history of abnormal signs of the breast. Getachew and colleagues suggested that individual beliefs, socioeconomic background and other factors caused delay in diagnosis [25]. As a result, breast cancer remained the most predominant type of cancer in the country [5,12,25].

Breast screening is well-accepted worldwide as a widely adopted means of early breast cancer detection [2,25]. Early detection could identify breast cancer at an early stage reducing poor clinical health. If cancer was diagnosed in an advanced state, it would lead to low survival rates [25,26]. Self-screening encourages women to check abnormal signs of their breasts regularly. If abnormal signs occurred, women would seek help for advanced examination faster [27]. According to a study by Darvishpour et al., whether one would perform breast cancer screening, is predicted by such factors as self-efficacy and perceived benefits, whereas perceived barriers reduced the likelihood of self-examination [22]. Khazir et al. suggested that having lower perceived barriers was associated with likelihood of participating in breast cancer early detection programmes [27]. According to the principle of HBM theory discussed earlier, if individuals had more exposure to screening behaviours, this would reduce perceived barriers to behaviours, and undertaking screening would be more likely [19].

It was revealed in our study that most participants did not feel confident that breast screening was the best means for early detection or prevention of advanced breast cancer prognosis. They believed that they could not control or avoid diseases and illnesses because it depended also on the visions of Allah and the rule of karma as a result of actions they had performed. Their life was dictated by Allah and karma. This is in line with Liamputtong and Suwankhong’s study [28]. Due to Allah’s will or one’s own karma, one had a chance of having breast cancer at any time without knowing or predicting this in advance. Although a woman might carry out regular breast examinations, it could not always prevent her far from getting breast cancer. Suwankhong and Liamputtong suggested in their study that religious belief plays an important part in how individuals decide about health practices or recognised determinants of breast cancer [29].

Many participants in this study paid little attention to seeking information and gaining knowledge about breast cancer. In addition, they showed less awareness about the risk of breast cancer. This may be because of a low perception of susceptibility to disease. According to the HBM, Rosenstock et al. and Skinner et al. articulated that perceived susceptibility is related to how individuals perceive a threat, and this predicts how individuals behave for their health [19,21]. It is important to note that, as this study was conducted during the COVID-19 pandemic, the pandemic had a huge influence on restricting the delivery of early breast cancer screening services. The healthcare system and healthcare professionals had to exert great effort to manage and control the pandemic. Regular campaigns and community activities to promote breast cancer prevention became a rare concern. As a result, women themselves also became less aware of the need to follow breast self-inspection plans. Getachew et al. pointed out that an uncertain situation increases the risk of breast cancer due to the delay in seeking health services or adhering to health prevention programmes as a matter of routine [25]. However, it was revealed in the study by Morman et al. that, if women were concerned about the risk of breast cancer development, they would seek information and breast examination programmes to promote their breast health [2]. This concern would affect awareness and increase concern to take responsibility for screening. Darvishpour et al. observed that perceived benefits increased the likelihood of breast self-examination [22]. It also boosted self-efficacy regarding such practice. Increase in perceived barriers reduced the likelihood of practicing breast examination. According to the HBM, having high self-efficacy and high perceived benefits and low perceived barriers would be associated with a high rate of self-examination performance.

Training for self-screening from health care providers is required regularly to ensure individuals’ skills and practice. We found that many women in our study had no confidence in self-examination. Skill training opportunities were organized by healthcare providers only occasionally. Receiving irregular training and follow-up reduces women’s awareness of breast cancer and likelihood of performing screening as part of their daily lives. This finding is in contrast to the findings of a study conducted by Haghbeen et al., who argued that individuals believed in the benefit of breast self-examination, they would have high level of awareness of prevention practice procedures and have fewer barriers to doing so. They would have increased self-efficacy for breast self-screening [5].

Beliefs and perceptions influence how individuals make decisions about health practices and behaviour [19]. Many participants in this study had knowledge about the severity of breast cancer, but they perceived that breast cancer mostly occurred in older women and was very rarely diagnosed among younger women. They did not often consider the danger, seriousness and complications of this disease among the younger generation. Thus, they paid relatively little attention to early detection or the importance of seeking further information to increase knowledge about breast health promotion and prevention. According to the principles of the health belief model, if individuals perceived risk and susceptibility for breast cancer, they would follow and maintain positive health practices [4,22]. However, this did not occur among younger women in our study.

Interestingly, we found a marked difference regarding religious beliefs between Muslim and Buddhist groups when the participants were asked about the likelihood of developing breast cancer in their life. Islam and Buddhism have influenced how people lead their lives and behave regarding their health. Muslim women in our study had a strong belief in the influence of God (Allah), whereas Buddhist women tended to refer to their own karma. Yew et al. described the important aspect of Islam as the belief in a God or Allah [30]. The deeds of individuals will be devoted to God, and this determines the life and destiny of each person. However, Thai Buddhists believe in the laws of karma, which refer to good deeds and bad deeds that a person has committed in the past that can have a consequence (good or bad) in later life.

### Implications for Healthcare, Strengths and Limitations

To our knowledge, this study is the first research project carried out in a rural community which considered a multicultural site in southern Thailand. The findings add to the literature in the area of health promotion and prevention, particularly with regard to women’s health. In rural communities in developing countries, there are insufficient healthcare facilities to support optimal health. The findings of our study could enable health professionals in the locality to be more aware that there are many at-risk groups of women in the rural community who do not pay sufficient attention to promoting breast health and preventing risk factors. Many barriers exist. This can increase the risk of developing advanced breast cancer among women in the nation. Thus, it is essential to pursue proactive actions to boost individual’s awareness of breast self-examination. The collaboration of all sectors in the community can strengthen breast health prevention and promote it as a key activity of the community. This could increase women’s awareness, leading to sustainable practice among at-risk groups of women.

The implications for future healthcare and research of the study include that healthcare settings in multicultural communities should pursue activities to enable at-risk women to have a platform to share their perceptions, beliefs and practices about breast cancer. These activities should be run either through health leaders in the community or village health volunteers (VHVs) to provide support, as well as to remind women about breast self-examination. These activities should be organised twice a year and become routine practice in healthcare settings. Moreover, it is important that means are provided for women to access health information using user-friendly technology and media as this can increase ease of access to necessary health information. Online applications, for example, are recognised as appropriate options in the 21st century as they involve use of real-time media, which can encourage people to take part in interactive communication for health consultation if needed. Such activities can be established based on individual health record data for disease prevention. Further research that uses quasi-experimental designs to develop health technology and media for women in multiethnic communities to increase their literacy and accessibility to breast health promotion and prevention is recommended.

However, the study has limitations. As a qualitative study, it provides insights and understanding about perceptions of breast cancer and screening prevention programmes amongst women, but the results of this study might not be applicable to women living in other social contexts in Thailand.

## 5. Conclusions

Breast cancer remains a public health problem in Thailand as it is the leading cause of death among Thai women today. Women in this study perceived that there were various factors causing breast cancer: genetics, individual behaviour, environment and religious beliefs. They perceived that a woman had a chance of developing breast cancer during their lifetime. Although they understood that early detection through breast self-examination was a basic technique to help check abnormal signs, most women were not confident that this could indicate breast cancer at an earlier stage. They had no confidence in performing breast self-examination. They trusted public healthcare workers at local health centres to perform this for them more often because of their professional skills. To prevent the risk of developing breast cancer among at-risk groups in the local community, regular breast self-examination to increase individual skills needed to be managed properly. This could be undertaken in parallel with regularly organised community activities in local areas. Public health providers need to be more concerned about perceptions, beliefs and practices regarding breast cancer and develop prevention practices that work better for women living in more diverse cultural locations so that they may be able to follow preventive practices and reduce their vulnerability to breast cancer.

## Figures and Tables

**Table 1:** The characteristics of research participants (n = 30).

Participant Characteristics	*n*	%
Age		
30–39	8	26.67
40–49	12	40.00
50–59	10	33.33
Mean, SD = 45.10, 8.25		
Education degree		
Primary school	10	33.33
Junior high school	9	30.00
Senior high school	7	23.33
Bachelor’s degree	3	13.33
Religion		
Buddhist	9	30.00
Muslim	21	70.00
Marital status		
Single	1	3.33
Married	25	83.33
Widowed	2	6.67
Divorce/Separated	2	6.66
Occupation		
Employee	6	20.00
Agriculture	20	66.67
Unemployed	4	13.33
Family history of cancer		
Yes	12	39.99
No	18	60.00
Abnormal history of breast		
Yes	4	13.33
No	26	86.67
Method used for screening		
Breast self-examination	23	76.67
Health personnel	5	16.67
Ultrasound	1	3.33
Mammogram	1	3.33
Menopause		
Yes	8	26.67
No	22	73.33
If yes, the age of menopause		
45–50	5	38.46
>50	8	61.54

## Data Availability

No data is available for this research.
